# β-diketone-cobalt complexes inhibit DNA synthesis and induce S-phase arrest in rat C6 glioma cells

**DOI:** 10.3892/ol.2013.1772

**Published:** 2013-12-24

**Authors:** KAIZHI ZHANG, XINGLI ZHAO, JUNZHI LIU, XIANGYANG FANG, XUEPENG WANG, XIAOHONG WANG, RUI LI

**Affiliations:** 1 China-Japan Union Hospital, Jilin University, Changchun, Jilin 130033, P.R. China; 2 Key Laboratory of Polyoxometalate Science of Ministry of Education, Northeast Normal University, Changchun, Jilin 130024, P.R. China

**Keywords:** β-diketone-cobalt complexes, DNA synthesis, glioma, cell cycle

## Abstract

β-diketone-cobalt complexes, a family of newly synthesized non-platinum metal compounds, exhibit potential antitumor activity; however, the antitumor mechanism is unclear. The current study investigated the mechanism by which β-diketone-cobalt complexes inhibit rat C6 glioma cell proliferation. It was found that β-diketone-cobalt complexes suppress rat C6 glioma cell viability in a dose-dependent manner (3.125–100 μg/ml). In rat C6 glioma cells, the IC_50_ value of β-diketone-cobalt complexes was 24.7±3.395 μg/ml and the IC_10_ value was 4.37±1.53 μg/ml, indicating a strong inhibitory effect. Further investigation suggested that β-diketone-cobalt complexes inhibit rat C6 glioma cell proliferation, which is associated with S-phase arrest and DNA synthesis inhibition. During this process, β-diketone-cobalt complexes decreased cyclin A expression and increased cyclin E and p21 expression. In addition, β-diketone-cobalt complexes exhibit a stronger antitumor capability than the antineoplastic agent, 5-fluorouracil.

## Introduction

Inorganic chemistry has its place in medicine, and metals, particularly transition metals, have various clinical applications (1,2). Cisplatin, as an inorganic antineoplastic agent, has been extensively used to treat tumors, but its clear side effects and tolerance limit its clinical applications. In addition, platinum complex with new ligands did not exhibit marked advantages in previous clinical trials. To date, only carboplatin and oxaliplatin have been used clinically (3,4).

β-diketone-cobalt complexes are polyoxometalates containing cobalt and traditional methods have been modified for the synthesis (5). β-diketone-cobalt complexes have been shown to suppress SMMC-7721 and SK-OV-3 tumor cell viability and interact with λ-DN (6); however, the molecular mechanisms of β-diketone-cobalt complexes against tumors remain unclear.

Brain glioma is a common central nervous system tumor, with at least five new cases per 100,000 individuals diagnosed worldwide each year (7,8). Malignant brain glioma extensively infiltrates normal brain tissues and is difficult to completely excise surgically. The relapse rate is high and conventional therapies used are radiotherapy and chemotherapy (9). Although present therapeutic methods are markedly advanced, the majority of patients cannot be cured (10). As present chemotherapeutics do not obtain ideal outcomes, the development of highly effective, low toxicity drugs for the treatment of brain glioma is required.

The current study focused on the effects of β-diketone-cobalt complexes against C6 rat glioma cell cytotoxicity and their potential molecular mechanisms of action against tumor cells.

## Materials and methods

### Antibodies and reagents

Anti-cyclin A, -cyclin E and -p21 polyclonal antibodies were purchased from Santa Cruz Biotechnology, Inc. (Santa Cruz, CA, USA), while GAPDH monoclonal antibody was purchased from Kangchen Bio-tech, Inc. (Shanghai, China). 3-(4,5-dimethylthiazol-2-yl)-2,5-diphenyltetrazolium bromide (MTT) was obtained from Sigma-Aldrich (St. Louis, MO, USA).

### Cell lines and culture

Rat C6 glioma cells were incubated in Dulbecco’s modified Eagle’s medium (Gibco Life Sciences, Grand Island, NY, USA) supplemented with 10% fetal bovine serum (Gibco Life Sciences), 2 mM L-glutamate, 100 U/ml penicillin and 100 μg/ml streptomycin at 37°C in a 5% CO_2_ incubator.

### MTT assay

Rat C6 glioma cells at 10^4^ cells/well were seeded onto 96-well plates for 24 h at 37°C and then treated with β-diketone-cobalt complexes. Next, 20 μl MTT solution (5 μg/ml) was added to each well and incubated for 4 h at 37°C prior to the removal of the culture medium. Dimethyl sulfoxide (150 μl) was then added and agitated for 10 min at room temperature. Absorbance values were measured at 570 nm using a Micro ELISA reader (Bio-Rad, Hercules, CA, USA). The inhibitory rate of β-diketone-cobalt complexes was calculated as the ratio of absorbance values of the experimental group to the control group. IC_10_ and IC_50_ values were calculated by SPSS version 19.0 (IBM, Armonk, NY, USA).

### Cell cycle analysis

Rat C6 glioma cells were seeded at a density of ~10^6^ cells/well in six-well plates at 37°C for 24 h. Cells were washed twice with ice-cold phosphate-buffered saline (PBS; pH 7.4), treated with β-diketone-cobalt complexes, fixed with 50% alcohol at 4°C overnight and then stained with propidium iodide (1 mg/ml) containing 1% RNAase A for 30 min. The cell cycle was analyzed using a flow cytometer (Epics XL ADC, Beckman Coulter, Miami, FL, USA).

### Western blot analysis

Rat C6 glioma cells were treated with β-diketone-cobalt complexes for 12 h prior to the preparation of cell lysates. Subsequently the cell lysates were separated through a 12% SDS-PAGE gel. Following electrophoresis, proteins were transferred to PVDF membranes, and blocked with 5% non-fat dry milk in TBST buffer (20 mM Tris-HCl pH 7.6, 150 mM NaCl and 0.05% Tween-20) for 1 h at room temperature. The membranes were subsequently probed with diluted primary antibodies in 1% milk/TBST at 4°C overnight, washed three times, incubated with HRP-conjugated secondary antibodies for 30 min at room temperature, and washed extensively prior to detection by chemiluminescence with the ECL-Plus kit (Beyotime, Haimen, China).

### [^3^H]-thymidine assay

Rat C6 glioma cell proliferation was quantified by [^3^H]-thymidine (GE Healthcare, Milan, Italy) incorporation, as described previously (12). Rat C6 glioma cells were seeded at a density of 10^6^ cells/well in six-well plates at 37°C for 24 h. Following treatment with β-diketone-cobalt complexes for 48 h, cells were incorporated with 20 μCi/ml [^3^H]-thymidine at 37°C for 2 h. Cells were then washed three times with PBS, lysed with 200 μl 4% trichloroacetic acid for 30 min and washed three times with 200 μl NaOH (0.1 M). The liquid was poured into a scintillating disc with the addition of 3 ml scintillation fluid. The counts per minute value was detected using a liquid scintillation counter (1450 MicroBeta TriLux, PerkinElmer Life Sciences, Boston, MA, USA).

### Cell morphology assay

Rat C6 glioma cells at 10^5^ cells/well were seeded onto 6-well plates for 24 h at 37°C and subsequently treated with β-diketone-cobalt complexes and 5-Fu. The cells were analyzed using a fluorescence microscope 48 h later. The images were acquired using an Olympus IX71 fluorescence microscope (Olympus, Tokyo, Japan)

### Statistical analysis

Experiments were repeated at least three times with four replicates per sample. Student’s t-test was used to calculate the statistical significance of the experimental results. P<0.05 and P<0.01 were considered to indicate statistically significant differences. Data are presented as the mean ± SD, unless stated otherwise.

## Results

### β-diketone-cobalt complexes suppress rat C6 glioma cell viability

**Figure 1:**
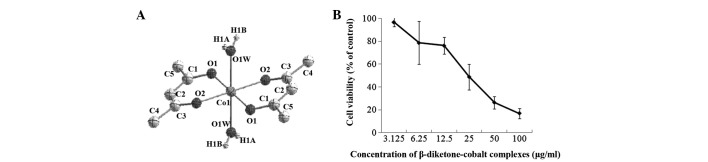
β-diketone-cobalt complexes suppress rat C6 glioma cell viability. (A) Structure of β-diketone-cobalt complexes. (B) Rat C6 glioma cells were treated with β-diketone-cobalt complexes (3.125, 6.25, 12.5, 25, 50 or 100 μg/ml) for 48 h and 0.1% (v/v) dimethyl sulfoxide served as negative control. Data are presented as the mean ± SD of three independent experiments, following 3-(4,5-dimethylthiazol-2-yl)-2,5-diphenyltetrazolium bromide.

The chemical formula of the β-diketone-cobalt complexes is Co(acac)_2_(H_2_O)_2_ [Co(acac)] and the structure is shown in Fig. 1A. The MTT results for rat C6 glioma cells following treatment with β-diketone-cobalt complexes (3.125, 6.25, 12.5, 25, 50 or 100 μg/ml) for 48 h demonstrated that β-diketone-cobalt complexes significantly suppress rat C6 glioma cell viability in a dose-dependent manner. In rat C6 glioma cells, the IC_50_ value of β-diketone-cobalt complexes was 24.7±3.395 μg/ml and IC_10_ value was 4.37±1.53 μg/ml (Fig. 1B). The abovementioned results revealed that β-diketone-cobalt complexes exhibit a marked inhibitory effect on rat C6 glioma cells.

### β-diketone-cobalt complexes inhibit rat C6 glioma cell proliferation

**Figure 2:**
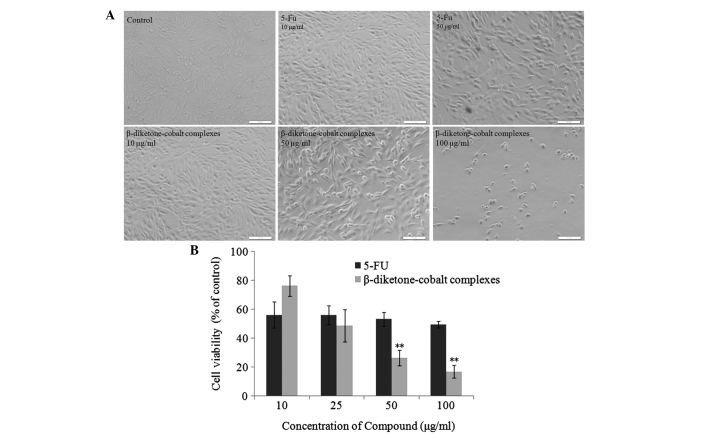
β-diketone-cobalt complexes inhibit rat C6 glioma cell proliferation. (A) Rat C6 glioma cells were treated with 10, 50 and 100 μg/ml β-diketone-cobalt complexes, and 10 and 50 μg/ml 5-Fu for 48 h. Images were captured under the light microscope (scale bar, 100 μm; magnification, ×200).(B) Rat C6 glioma cells were treated with 10, 25, 50 and 100 μg/ml β-diketone-cobalt complexes and 5-Fu for 48 hours. Dimethyl sulfoxide [0.1% (v/v)] served as a negative control. Data are presented as the mean ± SD of three independent experiments, following 3-(4,5-dimethylthiazol-2-yl)-2,5-diphenyltetrazolium bromide. ^**^P<0.01, vs. the same concentration of β-diketone-cobalt complexes and 5-Fu treatment groups. 5-Fu, 5-fluorouracil.

To understand the mechanisms by which β-diketone-cobalt complexes affect rat C6 glioma cell viability and its antitumor capacity, β-diketone-cobalt complexes were compared with an antineoplastic agent, 5-fluorouracil (5-Fu), *in vitro*. Firstly, rat C6 glioma cells were separately treated with β-diketone-cobalt complexes (10, 50 and 100 μg/ml) and 5-Fu (10 and 50 μg/ml) for 48 h. β-diketone-cobalt complexes and 5-Fu inhibited rat C6 glioma cell proliferation in a dose-dependent manner (Fig. 2A). Rat C6 glioma cells were analyzed using MTT assays following treatment with β-diketone-cobalt complexes and 5-Fu (10, 25, 50 and 100 μg/ml) for 48 h. With increased concentration, the inhibitory effects of β-diketone-cobalt complexes on rat C6 glioma cell proliferation were significantly stronger than those of 5-Fu (Fig. 2B). The abovementioned results revealed that β-diketone-cobalt complexes exert antitumor effects by inhibiting rat C6 glioma cell proliferation.

### β-diketone-cobalt complexes inhibit DNA synthesis and induce S-phase arrest in rat C6 glioma cells

**Figure 3:**
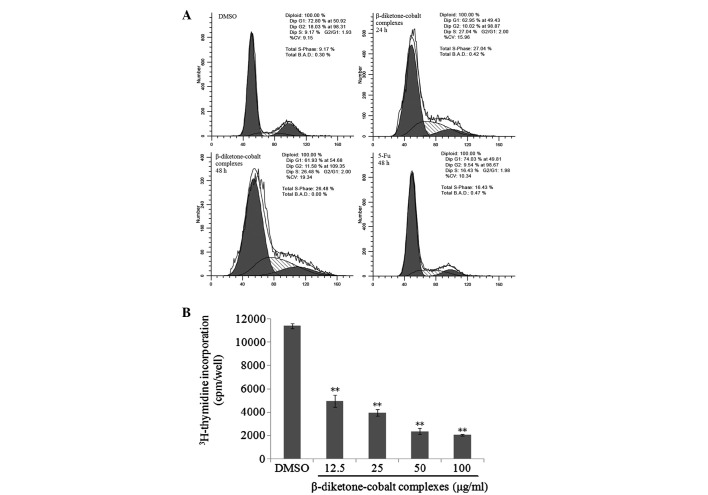
Effects of β-diketone-cobalt complexes on cell cycle. (A) Rat C6 glioma cells were treated with 25 μg/ml β-diketone-cobalt complexes for 24 and 48 h, and 25 μg/ml 5-Fu for 48 h. The relative number of cells within each cell cycle was determined by flow cytometry. (B) Rat C6 glioma cells were treated with 12.5, 25, 50 and 100 μg/ml β-diketone-cobalt complexes for 24 h, followed by [^3^H]-thymidine assay. ^**^P<0.01, vs. negative control. 5-Fu, 5-fluorouracil DMSO, dimethyl sulfoxide.

To further investigate the mechanisms by which β-diketone-cobalt complexes suppress rat C6 glioma cell proliferation, flow cytometry was utilized to identify the effects of β-diketone-cobalt complexes and 5-Fu on the rat C6 glioma cell cycle. Compared with the control group, the percentage of S-phase cells significantly increased from 9.17 to 27.04 and 26.48% following rat C6 glioma cell exposure to β-diketone-cobalt complexes for 24 and 48 h, respectively. The percentage of cells in S phase increased from 9.17 to 16.43% following exposure to 5-Fu for 48 h (Fig. 3A). Subsequently, [^3^H]-thymidine assay was employed to measure the effects of various concentrations of β-diketone-cobalt complexes on the DNA synthesis of rat C6 glioma cells for 24 h. Compared with the control group, with increased concentration of β-diketone-cobalt complexes, DNA synthesis in rat C6 glioma cells was evidently inhibited in a dose-dependent manner (Fig. 3B). It was concluded that β-diketone-cobalt complexes suppress rat C6 glioma cell proliferation by inhibiting DNA synthesis and inducing S-phase cell cycle arrest.

### Effects of β-diketone-cobalt complexes on cyclin A, cyclin E and p21 expression in rat C6 glioma cells

**Figure 4:**
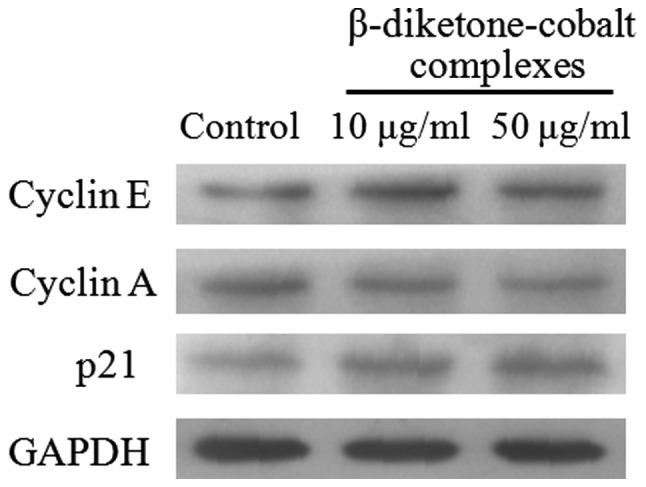
Effects of β-diketone-cobalt complexes on cell cycle-associated proteins. Rat C6 glioma cells were treated with 25 μg/ml β-diketone-cobalt complexes for 48 h and then cytoplasmic protein was extracted. The expression levels of cyclin A, cyclin E and p21 were detected by western blotting.

To identify proteins involved in S-phase arrest induced by β-diketone-cobalt complexes in rat C6 glioma cells, protein expression was detected in rat C6 glioma cells at 48 h following exposure to β-diketone-cobalt complexes. Results showed that β-diketone-cobalt complexes reduced cyclin A expression in rat C6 glioma cells (13), but increased cyclin E and p21 expression (Fig. 4).

## Discussion

β-diketone-cobalt complexes, newly synthesized non-platinum metal compounds, have been shown to inhibit SMMC-7721 and SK-OV-3 cell viability, but their antitumor mechanisms remain unclear (14). Brain glioma is a common tumor in the central nervous system and is difficult to completely excise surgically. Its relapse rate is high and conventional therapy is based on radiotherapy and chemotherapy; however, the outcomes of current chemotherapy drugs are not ideal. The present study first explored the mechanisms by which β-diketone-cobalt complexes inhibit rat C6 glioma cell proliferation and confirmed that β-diketone-cobalt complexes suppress rat C6 glioma cell viability in a dose-dependent manner (3.125–100 μg/ml). Of note, in rat C6 glioma cells, the IC_50_ value of β-diketone-cobalt complexes was 24.7±3.395 μg/ml and IC_10_ value was 4.37±1.53 μg/ml, showing a good inhibitory effect against tumors (Fig. 1B).

5-Fu, a common anticancer drug, is used for the treatment of head and neck cancer (15). 5-Fu interacts with nucleic acid metabolism, leading to cytotoxicity and cell death, thus, exerting its antitumor activity (16,17). Following comparison, the current study confirmed that β-diketone-cobalt complexes exhibit marked antitumor activity *in vitro* compared with 5-Fu (Fig. 2A). β-diketone-cobalt complexes at low concentrations significantly inhibited DNA synthesis in rat C6 glioma cells (Fig. 3B). Whether β-diketone-cobalt complexes, similar to conventional chemotherapy drugs, are simple cytotoxic drugs is poorly understood. The present study revealed that the inhibitory effect of β-diketone-cobalt complexes on rat C6 glioma cell proliferation correlates with S-phase arrest (Figs. 2A and 3A). However, 5-Fu did not suppress cell proliferation by cell cycle arrest (Fig. 3A), in contrast to the antitumor mechanisms of β-diketone-cobalt complexes.

Cell cycle regulation depends on two protein families, the cyclins and cyclin-dependent protein kinases (CDKs). During the cell cycle, cyclin expression dynamically alters and during the transition from G1 to S phase, cyclin E activates CDKs and cyclin E expression increases. Cyclin E expression is downregulated after entering S phase (18,19). In the current study, cyclin E expression increased at 24 h following treatment with β-diketone-cobalt complexes and diminished at 48 h (Fig. 4). Cyclin A plays a key role in S phase, but p21 causes cell cycle arrest by inhibiting CDK activity (20–22). However, β-diketone-cobalt complexes decreased the expression levels of cyclin A and p21 (Fig. 4). In conclusion, β-diketone-cobalt complexes significantly suppress rat C6 glioma cell proliferation, showing a potential ability for the development of novel antitumor drugs.
